# The Season of Appeals

**Published:** 1890-12-27

**Authors:** 


### PASSING TOPICS.

### THE SEASON OF APPEALS.

At this season of the year, a reputation for benevolence is apt to cause its possessor considerable inconvenience. At no time, perhaps, does it add to his comfort, but now especially, when Christmas and the New Year lend licence to importunity, his life becomes a burden and his happiness a delusion. Always the receptacle of much that is distressing, his sensibilities now are charged to repletion. Everybody with a trouble lays it at his feet. Tales of want and woe break upon his ear unceasingly, like waves upon a sandy shore, and to the utmost of his capacity he absorbs their melancholy volume until he himself is saturated with a sea of tears. Nor is this all. Added to the personal demands of his poorer neighbours, and maybe his impecunious relations, come the appeals of the institutions, and as his eye glances over the morning papers, a legion of philanthropic undertakings invite his aid and increase his perplexity. Though he be the most generous soul alive and rich to boot, he cannot give to all; what wonder if now and again, in a revulsion of feeling brought about by sheer weariness and vexation, he decides to give to none?

To such a one, the target for every shaft, it is impossible to offer protection from attack; it is difficult to suggest even means of defence, but we may perhaps help to put him upon his guard against some of the more specious of the onslaughts made upon his purse—those for objects ostensibly allied with medical charity. It is perfectly well-known to the well-read in such matters that there are nigh upon a hundred so-called hospitals in the Metropolis; it is but imperfectly known to the benevolent that the extinction of say, one-half of them, might be viewed with composure, and that in all that constitutes true value and usefulness the remainder would become under these changed conditions more than equal to the whole. But the law which protects the public against the quack practitioner takes no heed of the quack hospital, and the liberty of the subject includes the liberty to impose upon the benevolent.

It may seem strange, and to the uninitiated, almost incredible that an appeal for an undertaking perhaps patronized by Royalty itself and boasting a string of aristocratic followers should be open to question. But so it not seldom is, and unless the institution be too well known, as in the case of most of our great and really useful hospitals, to render the precaution unnecessary, it would be well if those appealed to would carefully scrutinise the subject matter put before them, and by the aid of a few simple rules, or with the help of their medical friends, learn to discriminate between the merits of the several applicants for a share in their bounty.

We would strongly urge that no unknown hospital ought to be assisted which fails to give ample proof of its *bona fides* by supplying, for instance, a plain statement of the work performed, including the number of beds maintained, and the amount of the normal expenditure, with accounts audited and certified by a public accountant. The number and status of the members of the active staff furnish perhaps the surest test of the character and utility of a special hospital, and a full list of the contributors of the past year, with details of their contributions and the total duly carried to the statement of receipts and expenditure, is a sure protection against the *sub-rosâ* deduction of those enormous percentages upon sums received, which appear essential to the existence of worthless institutions. Every donor should remember that when he makes a gift to an undeserving institution, he is not only wasting money sadly needed by many indispensable charities, but he is actively co-operating with those whose instincts are predatory rather than philanthropic.

Agricola.

